# Cox Model Setup: Garshick et al. Respond

**DOI:** 10.1289/ehp.1205573R

**Published:** 2012-10-01

**Authors:** Eric Garshick, Francine Laden, Jaime E. Hart, Mary E. Davis, Ellen A. Eisen, Thomas J. Smith

**Affiliations:** 1 Pulmonary and Critical Care Medicine Section, VA Boston Healthcare System, Harvard Medical School, Boston, Massachusetts, E-mail: Eric.Garshick@va.gov; 2 Channing Division of Network Medicine, Brigham and Women’s Hospital, Harvard Medical School, Boston, Massachusetts; 3 Department of Urban and Environmental Policy and Planning, Tufts University, Medford, Massachusetts; 4 Environmental Health Sciences Division, School of Public Health, University of California, Berkeley, Berkeley, California; 5 Exposure, Epidemiology and Risk Program, Department of Environmental Health, Harvard School of Public Health, Boston, Massachusetts

We appreciate the interest in our article (Garshick et al. 2012). In his letter, Morfeld suggests that the analytic approach used for Cox proportional hazard regression modeling included two similar adjustments for year of birth. We disagree with this comment.

In the analysis, risk sets were generated using attained age as the timeline. An ordinal variable for calendar year was included as a covariate; thus, we do agree that our approach adjusted for exact year of birth.

We also stratified the analysis on decade of hire (four groups) and age in 1985 (four groups). We stratified on decade of hire to adjust for different unmeasured work practices and vehicle characteristics. We stratified on age in 1985 because the age at which persons enter the study is a determinant of lung cancer risk; participants had to be healthy enough to remain employed to enter the cohort in 1985. Two of the survival curves for decade of hire overlap unless they are jointly stratified by age in 1985, indicating that joint stratification is important to maintain the proportional hazards assumption. This approach allows us to maintain the assumption of proportional hazards and to finely adjust for lung cancer secular trends and attained age but does not adjust twice for year of birth within the same model.

Our analytic approach also included sensitivity analyses with and without total years of employment as a time-dependent covariate to assess its effect as a potential confounder. Morfeld suggests that adjusting cumulative exposure by duration of employment time reduces cumulative exposure to an estimate of long-term average concentration. We agree that if exposure in our workers was relatively constant, cumulative exposure would be the simple product of duration and average exposure. However, exposure varies considerably over time and between and within jobs. Therefore, it is not surprising that the results for duration and average exposure are not similar to those for the cumulative exposure.

In his letter, Morfeld states that “an adjustment of cumulative exposure by total duration of employment should not be confused with an approach adjusting for the healthy worker survivor bias.” However, our assessment (Garshick et al. 2012) identified years of employment as a negative confounder because it was positively associated with cumulative exposure and negatively associated with lung cancer risk. Failure to account for this would result in the underestimation of lung cancer risk. Adjustment for total duration of employment strengthened effects with cumulative exposure and may be considered an assessment of the effects of cumulative exposure at varying durations of employment.

Because lung cancer risk decreased with total employment duration, we can treat duration as a surrogate of time-varying health status. As we noted in our article (Garshick et al. 2012), “this was likely due to bias caused by left truncation in a cohort composed of prevalent hires combined with a healthy worker survivor effect.” We were not surprised to note this relationship because of the structure of the cohort. As shown previously by Applebaum et al. (2011), left truncation results in downward bias with exposure duration. In our article (Garshick et al. 2012), we extensively discussed a healthy worker survivor effect and left truncation and also cited studies where these effects have been observed. We also cited examples where adjustment for work duration was used as a method to address bias due to a healthy worker survivor effect.

As Morfeld noted in his letter, adjustment for the healthy worker survivor effect is complex. We do not claim that adjustment using employment duration completely adjusts for a healthy worker survivor effect, but our results provided evidence that it is present in this cohort and should be addressed.
